# Ghrelin peptide in heart failure: when is showtime?

**DOI:** 10.3389/fcvm.2025.1574521

**Published:** 2025-07-04

**Authors:** Som P. Singh, Anand Chockalingam

**Affiliations:** ^1^ Department of Internal Medicine, University of Texas Health Sciences Center at Houston, Houston, TX, United States; ^2^ Department of Cardiology, Charleston Area Medical Center, Charleston, WV, United States

**Keywords:** Ghrelin, heart failure, biomarker, inotrope, cachexia

## Introduction

Ghrelin has built a profile as a peptide hormone critical in appetite and adiposity metabolic pathways, and it is associated as a ligand for growth hormone secretagogue receptors (1). It is largely produced in the stomach and small intestine, then secreted into the bloodstream. However, there are also reports of ghrelin production outside the gastrointestinal system, including in vital organs such as the kidney and the lung (2, 3). The active structure of ghrelin has largely been identified as a 28-amino-acid peptide. There is an n-octanoyl group attached to serine at position 3 that has been demonstrated to play a key role in its ability to bind to the growth hormone secretagogue receptors and function in numerous physiological processes, including growth hormone release and appetite (4).

Additionally, a growing body of literature has observed its role in cardiovascular pathophysiology (5, 6). Ghrelin has been demonstrated to be expressed by human cardiomyocytes on culture medium (7). Moreover, growth hormone secretagogue receptors have also been found to be localized in cardiac tissue, including in the myocardial and smooth muscle cells (8, 9). Among the effects of ghrelin in cardiovascular diseases, experimental studies have demonstrated a reduction in the incidence of ventricular tachyarrhythmia in a mouse myocardial infarction model (10). Moreover, experimental evidence also suggests that des-acyl ghrelin had beneficial cardiovascular effects in the settings of doxorubicin-induced cardiomyopathy via limiting cardiac fibrosis and cellular apoptosis (11).

**Figure 1:**
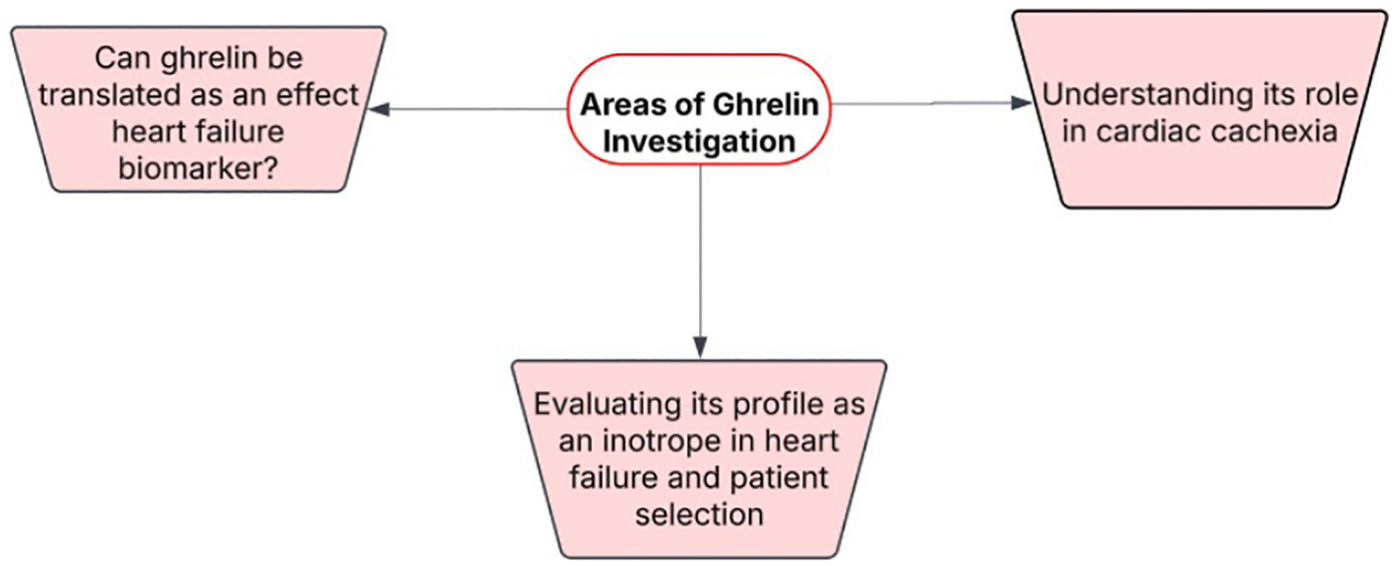
Areas of investigation regarding ghrelin in heart failure.

There is also a growing body of literature is investigating ghrelin as a key target in heart failure (6). As interest in this peptide hormone rises leading to new pre-clinical and clinical data to emerge, there is a need to examine key areas and highlight future areas for further investigation. This paper highlights three key areas of ghrelin in heart failure and discusses what should be further investigated (Figure 1).

### Ghrelin as an inotrope

The use of inotropic agents in patients with heart failure has been seen in the inpatient setting, such as those with low cardiac output, hypotension, and end-organ dysfunction (12). They have also been used as hemodynamic optimization therapy to bridge to heart transplantation or prior left ventricle assist device (LVAD) implantation and in palliative settings (12–14). A key physiological effect of ghrelin is its ability to modulate cardiac contractility, functioning as a positive inotrope. However, the exact mechanisms of how this is performed by ghrelin continue to be investigated in pre-clinical and clinical studies. One of the earliest randomized trials where human synthetic ghrelin infusion was provided to individuals with clinical congestive heart failure (CHF) was by Nagaya et al. in 2001 (15). This study found that the group of individuals with CHF receiving ghrelin infusion had a statistically significant increase in stroke volume and cardiac index. Many years later, a randomized trial by Lund et al. in 2023 with 31 patients with heart failure with reduced ejection fraction, where individuals with human synthetic ghrelin infusion were provided to patients, and this group had a statistically significant 28% increase in cardiac output and 14.5% increase in stroke volume (16). Lund et al. also demonstrated an experimental *ex vivo* study were isolated mouse cardiomyocytes, with the administration of acyl ghrelin, increased load-dependent contractility. However, this occurred independent of preload or afterload, and most importantly, without affecting cellular calcium mobilization (16). This mechanism may decrease the risk of complications such as tachycardia, arrythmia, hypotension and cardiac ischemia that limit use of established inotropic agents (17, 18).

Additionally, ghrelin has been demonstrated to have protective effects in cardiovascular remodeling, which provides an advantageous profile as an inotrope. However, these cardioprotective effects are still not well understood. Eid et al. reported an animal model that suggested that the activation of JAK2/STAT3 signaling and inhibition of STAT1 signaling inhibited cardiac myocyte apoptosis during cardiovascular remodeling (19). Mao et al. also reported that these effects contribute to a cholinergic anti-inflammatory process in cardiac hypertrophy (20). Ghrelin has also demonstrated decreased sympathetic activity as well as anti-arrhythmic effects in subjects following myocardial infarction (10, 21).

Currently available inotropic agents for clinical practice are used for acute hemodynamic benefits despite increasing mortality (22). As the evolving literature on metabolics suggests, ghrelin infusion may not increase cardiac mortality in acute heart failure, this could be a key area of future investigation. When present, right heart failure significantly worsens clinical outcomes. Emerging data among individuals with heart failure with reduced ejection fraction (HFrEF), treatment with acyl ghrelin group improves right ventricular-pulmonary arterial coupling compared to placebo (23). This is key as increases in cardiac output could physiologically disrupt right ventricular-pulmonary arterial coupling in the setting of HFrEF and impair right ventricular function more (24). These findings are promising, and future investigations could evaluate ghrelin for managing acute heart failure instead of currently established cardiac inotropic therapies.

### Ghrelin as a heart failure biomarker

Biomarkers can aid in providing risk and severity data and guide therapeutic management for patients with heart failure (25). In addition to positive inotropy, ghrelin may also serve as a biomarker for individuals with heart failure and has even been explored as a potential biomarker in diabetic cardiomyopathy (26, 27). Yuan et al. reported a cohort of 241 individuals with advanced heart failure, and this study found a statistically significant positive correlation between ghrelin concentration and NT-proBNP levels and heart failure severity (28). However, there needs to be further investigation to better understand the behavior profile of ghrelin in heart failure, as the findings by Chen et al. demonstrated that plasma ghrelin levels were significantly lower in their cohort of patients with CHF compared to controls and correlated inversely with plasma NT-proBNP levels (29). Moreover, the utility of ghrelin as a biomarker must be explored by future investigations to determine if it guides therapeutic decisions in heart failure. Ghrelin levels may be affected by heart failure stage, comorbidities, and by sampling postprandial, as in some of the above studies. Overall, ghrelin may serve as a new biomarker that aids clinicians in creating a more personalized management plan for their patients (30).

### Ghrelin and cardiac cachexia

The presence of cardiac cachexia is linked to considerable morbidity in heart failure (31, 32). Nagaya et al. reported that participants with CHF and cardiac cachexia demonstrated high plasma ghrelin levels (33). Moreover, there is literature that suggests that there may be resistance to appetite stimulation by ghrelin in heart failure, and this may be a compensatory response in cardiac cachexia (34, 35). The presence of cardiac cachexia may be a key selection factor for which patients may optimally benefit from exogenous ghrelin administration as well as advanced heart failure therapies (36). This makes it imperative for further trials to be designed that observe the effects of exogenous ghrelin administration on cardiac contractility and output for patients with CHF associated with cardiac cachexia and those without cardiac cachexia. Likewise, this makes it critical in developing studies that observe the role of ghrelin as a biomarker for patients in guiding treatment plans.

## Conclusion

Pre-clinical and clinical studies demonstrate key promise for ghrelin as a heart failure biomarker and an inotropic agent, given its prior demonstration of increased cardiac contractility and inhibition of cardiac myocyte apoptosis during cardiovascular remodeling. This raises attention to the need to investigate further which patients are most appropriate for this inotropic administration in larger cohorts. This is critical considering the numerous organ systems upon which ghrelin and growth hormone secretagogue receptors are involved in. Additionally, there is a paucity of data observing the long-term cardiovascular effects of ghrelin administration and cardiac cachexia. However, this paper is not without its limitations as well. This paper aims to highlight critical data on ghrelin in heart failure metabolics but does not encompass the complete potential of this hormonal peptide in heart failure.
